# Brain Metastasis in the Emergency Department: Epidemiology, Presentation, Investigations, and Management

**DOI:** 10.3390/cancers16142583

**Published:** 2024-07-19

**Authors:** Marianne Zoghbi, Mohammad Jad Moussa, Jim Dagher, Elio Haroun, Aiham Qdaisat, Emad D. Singer, Yara E. Karam, Sai-Ching J. Yeung, Patrick Chaftari

**Affiliations:** 1 Department of Leukemia, The University of Texas MD Anderson Cancer Center, Houston, TX 77030, USA; mzoghbi@mdanderson.org; 2 Department of Genitourinary Medical Oncology, The University of Texas MD Anderson Cancer Center, Houston, TX 77030, USA; 3 Faculty of Medicine, Saint Joseph University of Beirut, Beirut 1100, Lebanon; 4 Department of Emergency Medicine, The University of Texas MD Anderson Cancer Center, Houston, TX 77030, USA; 5 Division of Cancer Prevention and Population Sciences, The University of Texas MD Anderson Cancer Center, Houston, TX 77030, USA; 6 Department of Behavioral Sciences, The University of Texas MD Anderson Cancer Center, Houston, TX 77030, USA

**Keywords:** brain metastases, cancer, emergency department, epidemiology, presentation, investigation, management

## Abstract

**Simple Summary:**

Brain metastases (BMs), the most common type of cerebral tumor, are primarily associated with lung cancer, breast cancer, and melanoma. Patients typically present to the emergency department (ED) with insidious symptoms such as headaches, focal neurological deficits, seizures, and signs of increased intracranial pressure. Key symptomatic treatment in the ED includes corticosteroids to manage peritumoral edema, and carefully selected antiepileptic medications for tumor-related epileptic conditions. After stabilization, the treatment philosophy for BMs should prioritize effective yet minimally toxic options to maximize quality of life. Surgery is recommended for accessible BMs in patients with good performance status. Radiation therapy and systemic treatments, including chemotherapy, targeted therapy, and immunotherapy, are available options for managing disease progression. Given that over half of patients with BMs in the ED are admitted, a better understanding of avoidable hospitalizations is essential to differentiate those who can be safely discharged from those needing observation or hospitalization.

**Abstract:**

Brain metastases (BMs) are the most prevalent type of cerebral tumor, significantly affecting survival. In adults, lung cancer, breast cancer, and melanoma are the primary cancers associated with BMs. Symptoms often result from brain compression, and patients may present to the emergency department (ED) with life-threatening conditions. The goal of treatment of BMs is to maximize survival and quality of life by choosing the least toxic therapy. Surgical resection followed by cavity radiation or definitive stereotactic radiosurgery remains the standard approach, depending on the patient’s condition. Conversely, whole brain radiation therapy is becoming more limited to cases with multiple inoperable BMs and is less frequently used for postoperative control. BMs often signal advanced systemic disease, and patients usually present to the ED with poorly controlled symptoms, justifying hospitalization. Over half of patients with BMs in the ED are admitted, making effective ED-based management a challenge. This article reviews the epidemiology, clinical manifestations, and current treatment options of patients with BMs. Additionally, it provides an overview of ED management and highlights the challenges faced in this setting. An improved understanding of the reasons for potentially avoidable hospitalizations in cancer patients with BMs is needed and could help emergency physicians distinguish patients who can be safely discharged from those who require observation or hospitalization.

## 1. Introduction

Brain metastases (BMs) are the most prevalent type of cerebral tumor. Their considerable impact on morbidity and patient survival makes BMs a dreadful complication of systemic cancers, with overall 2-year survival percentages not exceeding single digits [1]. The poor prognosis of patients who develop BMs can be attributed either to uncontrolled primary cancer or to neurological complications of metastases. Furthermore, the prevalence of BMs is increasing, likely due to advances in diagnostic procedures and primary cancer treatment [2]. 

Most symptoms triggered by BMs arise from the mass effect on the brain, resulting in specific focal deficits depending on the location of the metastasis, as well as substantial morbidity in patients [3]. Optimal management of BMs has remained a challenge for years. Current treatments revolve around surgery, radiation therapy (RT), and chemotherapy. However, targeted therapies are emerging and boosting the management of BMs. With these advances in treatment options, an increasing number of patients are experiencing positive outcomes, even within palliative care settings. For this reason, more studies are integrating patients with BMs, because management of BMs is key to improving quality of life (QoL) [4]. 

It is important to recognize that over half of patients with BMs presenting to the emergency department (ED) are admitted to the hospital [5], and effective ED-based management of these patients remains a challenge. Given that the prognosis of patients with BMs is constantly evolving, there is a critical need to improve ED care, which will affect the subsequent course of patient care.

This article seeks to improve understanding of the contemporary approach to managing BMs by reviewing the epidemiology, clinical manifestations, and current treatment options for BMs. Additionally, this article provides an overview of the workup and management of BMs, focusing on ED disposition and highlighting the challenges that EDs face with this patient population.

## 2. Epidemiology

The incidence of intracranial malignancies, which involve BMs and leptomeningeal disease, has been on the rise, attributed to enhanced screening methods and prolonged survival with advanced systemic therapy [6]. However, another contributing factor to the increased incidence of BMs could be the limited ability of new cancer treatments to cross the blood–brain barrier, making the central nervous system (CNS) a “sanctuary” site for metastasis [7]. BMs represent the most common intracranial malignancies [3] and are ten times more common than primary brain cancers [7]. About 10% of cancer patients develop BMs during their illness [8], and up to 37% of patients also develop leptomeningeal disease [9]. At presentation, up to 12% of patients have simultaneous BMs and leptomeningeal disease [9].

In children, brain metastasis is rare, with most (61.3%) originating from primary neuroblastoma, followed by non-CNS sarcomas (16%) [10]. In adults, the most common primary cancers with a high potential for intracranial metastasis include lung cancer, breast cancer, and melanoma. BMs are reported in up to 56% of patients with lung cancer of any type, followed by approximately 20% of patients with breast cancer, and up to 16% with melanoma [4]. Non-small-cell lung cancer (NSCLC) is the most common subtype of lung cancer, accounting for up to 44% of BMs from lung cancer, with adenocarcinomas contributing up to 50% of NSCLC BMs [11]. Small-cell lung cancer (SCLC) accounts for only 6–18% of BMs from lung cancer but is more aggressive, with up to 20% of patients with SCLC having BMs at diagnosis and approximately 50%–80% of patients developing BMs over the course of the disease [11]. About two-thirds of BMs from breast cancer are associated with the triple-negative breast cancer subtype (i.e., negative for estrogen and progesterone receptors, and no HER2 amplification) and the HER2-positive subtype [12]. BMs can also arise from renal, prostate, ovarian, uterine, gastrointestinal, hepatic, pancreatic, adrenal, thyroid, and bone cancers [13]. Approximately 25% of patients with melanoma or lung cancer and up to 10% of patients with breast cancer or renal cell carcinoma may have BMs at the time of diagnosis [12]. Most BMs originate in the cerebral hemispheres (accounting for 75%), followed by the cerebellum (21%) and the brain stem (3%) [14].

BMs are associated with elevated morbidity and mortality [15], with a historic median survival duration of 3.6 to 3.8 months following diagnosis [16]. The estimated 2-year and 5-year overall survival rates across all primary tumor types are 8.1% and 2.4%, respectively [1]. Studies have shown that around 52% of patients with BMs die of neurological disease [17]. Patients diagnosed with BMs and primary prostate cancer, bronchoalveolar carcinoma, or breast cancer have exhibited the longest median overall survival durations, at 12 months, 10 months, and 10 months, respectively [18]. 

A retrospective study by Lamba et al. examined ED visits and hospitalizations among older patients with BMs. The study showed an average of 2.8 ED visits per person-year and two hospitalizations per person-year. Acute respiratory failure was the leading cause of ED visits, accounting for 5.4% of the visits. Other common reasons for ED visits included pneumonia (4.5%), lung cancer (4.1%), sepsis (3.8%), malaise and fatigue (3.4%), and intracranial disease (3.3%). Regarding hospitalization within 1 day of an ED visit, 58.2% of first ED visits per patient resulted in inpatient admission with shortness of breath as the primary reason for hospitalization, representing 6.2% of admissions. Other common reasons for admission were malaise and fatigue (5.9%), pneumonia (5.4%), altered mental status (4.8%), and lung cancer (3.4%) [19].

## 3. Presentation at the ED

### 3.1. Clinical Presentation

Clinical presentation can vary, with BMs typically exhibiting an insidious evolution, often prompting patients to seek medical attention only after symptoms arise [20]. Most symptoms stem from brain compression and increased intracranial pressure resulting from expanding tumor size or edema [3]. 

Headache is the most commonly reported symptom, in about half of patients [21], with associated papilledema observed in 15–25% of cases [2]. Focal neurologic symptoms are also a common presentation of BMs, occurring as the first manifestation in up to 40% of patients [3]. Additionally, seizures are a major cause of morbidity in patients with BMs, occurring in 15–25% of patients [22,23]. Seizures are less common in patients with BMs than in those with primary brain neoplasms [22]. Focal symptoms and seizure risk depend on the tumor location, presence of tumoral hemorrhage, and primary tumor pathology [3]. In one large retrospective dataset of patients with BMs, 24% reported tumor-related seizures [24]. Seizures were observed in 67% of patients with melanoma, making melanoma the primary cancer with the highest frequency of seizures, possibly due to the tendency for intracranial bleeding in BMs from this cancer type [24].

The presentation and manifestation of seizures may vary from grand mal (tonic–clonic seizure) to focal nonconvulsive seizure, such as absence seizure or occipital lobe seizure (perception of light flashes). Patients may report to the ED with life-threatening conditions such as status epilepticus (SE). SE remains a medical emergency, with 31.6% of patients dying within 30 days of developing this condition. High mortality rates are not directly attributed to SE but rather indicate an overall poor patient status in which SE develops [25]. Possible provoking factors for SE include increased size of metastases, hemorrhage, infection, or metabolic abnormalities [25]. 

Only 36.8% of patients who experience SE have seizures after being diagnosed with BMs. The most common subtype of SE is focal nonconvulsive SE [25]. The most common types of nonconvulsive SE are absence SE (ASE) and complex partial SE, primarily manifesting as altered mental status that ranges from confusion to coma [26]. Practitioners should suspect nonconvulsive SE in patients who present with a sudden onset of fluctuating mental status and faint clinical signs such as lip smacking and eye twitching [26]. Moreover, Todd paralysis can be observed following partial seizures or generalized tonic–clonic seizures [27]. Depending on the type of seizure and whether the patient has sustained cortical structural damage, the paralysis can last from minutes to days [28].

Some patients may experience acute symptoms mimicking a stroke due to intratumoral hemorrhage, particularly common in metastasis from melanoma or renal cell carcinoma, which have a higher risk of hemorrhage [2]. It has been reported that hemorrhagic brain metastasis is the most common type of spontaneous intracranial hemorrhage among cancer patients presenting to the ED, accounting for one-third of cases [29]. Although intratumor bleeding including intra-metastatic bleeding has better survival outcomes (i.e., in-hospital mortality and 7-day mortality) when compared to other types of intracranial hemorrhage for cancer patients presenting to the ED [29], the presentation and workup in the ED are similar. Patients may also present with aphasia, ataxia, altered mental status, somnolence, cognitive decline, personality changes, and visual disturbances [3,30]. The development of BMs significantly affects the QoL and prognosis of patients [20]. Thus, the emergence of new neurologic symptoms in cancer patients should raise suspicion for BMs and rapidly encourage neurological investigations to ensure timely access to targeted therapies [2]. Given the broad differential diagnosis of critical neurological conditions, a brain computed tomography (CT) scan is the most commonly used immediate imaging choice for cancer patients presenting with emergent non-traumatic neurological symptoms in the ED [31]

### 3.2. Symptomatic Treatment at the ED

#### 3.2.1. Peritumoral Edema 

Corticosteroids are commonly used alongside pain medication to effectively alleviate symptoms associated with peritumoral edema [32]. However, potential side effects of corticosteroids, including weight gain, delayed wound healing, hyperglycemia, and mood swings, require tapering as soon as symptoms improve.

The standard agent used to treat symptoms of peritumoral edema is dexamethasone. The initial dose should be determined by the severity of symptoms, because this agent exhibits a dose-dependent anti-edema effect. Symptoms may be generalized (e.g., headache, nausea, vomiting) or focal (e.g., aphasia, hemiparesis) depending on tumor location and extent of the edema. The standard approach is as follows [32,33,34]:

- Patients with mild symptoms, such as mild headache, can benefit from 2 to 4 mg of dexamethasone orally once or twice daily.
- Patients with moderate to severe symptoms such as severe headache, vomiting, seizures, and significant focal deficits require a 10 mg loading dose of intravenous dexamethasone followed by an initial maintenance dose of 8 to 16 mg daily in 1 to 4 divided doses orally (or intravenously for patients not tolerating oral medications).
- Patients with a significant increase in intracranial pressure, leading to drowsiness and other signs of impending herniation, require prompt management. A 10 mg intravenous bolus dose of dexamethasone should be given, followed by 16 mg/day in 2 to 4 divided doses. Acutely, up to 40 mg of dexamethasone per day can be tolerated as a maintenance dose for severe mass effect symptoms. Hypertonic saline and mannitol can also be considered to help control increased intracranial pressure.

#### 3.2.2. Tumor-Related Epilepsy

Symptomatic management of tumor-related epilepsy should take into consideration patients’ characteristics, comorbidities, and other concurrent therapies. Following evidence-based recommendations, practitioners should avoid giving CYP3A4 coenzyme-inducing antiepileptic drugs, such as carbamazepine, phenytoin, and phenobarbital, because these drugs may interfere with chemotherapy. For that reason, the antiepileptic drugs of choice are levetiracetam and valproic acid [35].

Levetiracetam has no significant drug interactions, but about 5% of patients develop irritability, aggression, or psychosis, necessitating discontinuation [22]. Valproic acid is also well tolerated, although it may cause increased appetite and trembling. The primary concern, however, is dose-dependent thrombopenia, which can affect simultaneous chemotherapy [36].

If anticonvulsant monotherapy is not sufficient to control seizures, a combination of levetiracetam and valproic can be offered. A retrospective analysis showed seizure control in 77.7% of patients receiving levetiracetam alone, 69.5% in patients receiving valproic acid alone, and 60.3% in patients receiving both drugs [37]. If these are still insufficient, options include adding lacosamide, lamotrigine, or zonisamide, based on their efficacy and tolerability in patients with tumor-related epilepsy [38,39].

An example of a medication schedule for patients is 250 mg of levetiracetam twice per day for the first week, followed by 500 mg twice per day to achieve a therapeutic plasma range of 5–25 mg/L. If seizures are not controlled, valproic acid can be added at a dose of 20–25 mg/kg per day, aiming for a therapeutic range of 50–100 mg/L [22]. 

The definitive treatment to control tumor-related epilepsy remains surgical resection, when possible [40]. Another consideration is whether to start antiepileptic drug prophylaxis in patients presenting to the ED with BMs and no prior history of seizures. Current recommendations are against long-term antiepileptic drug prophylaxis in patients with newly diagnosed brain tumors, including those with BMs [35]. 

#### 3.2.3. Status Epilepticus

The most commonly used treatments to control SE are benzodiazepines and levetiracetam [25]. The first-line therapy for nonconvulsive SE is intravenous benzodiazepines, followed by intravenous phenytoin or fosphenytoin. Using valproic acid as a second-line treatment is also reasonable, especially with absence SE. Electroencephalographic monitoring should be conducted during treatment to identify the therapeutic goal [26]. The duration of SE in patients with BMs is extremely variable; however, SE will resolve in most patients with appropriate treatment [25].

The options for symptomatic treatment mentioned above are summarized in Table 1.

## 4. Investigations and Clinical Workup

The approach to investigating BMs depends on two key factors: presentation (symptomatic and asymptomatic) and timing of symptoms (acute, subacute, or chronic). Thus, the best place for health care providers to start is with a detailed history and clinical examination, with a particular emphasis on neurological assessment. Although BMs signal an underlying neoplastic process de facto, the differential diagnosis in any clinical scenario starts with the presenting symptoms. For example, in the ED, the differential diagnosis for suspected BMs in cancer patients is broad enough to exclude, by order of priority, cerebral infarction or hemorrhage, infectious diseases, cancer treatment-related effects (e.g., effects of RT or systemic therapies), and miscellaneous paraneoplastic phenomena. This section focuses on discussing state-of-the-art medical techniques for diagnosing BMs, regardless of the presenting context. Two specific situations merit attention in the workup: the efficacy of imaging in determining the number and locations of BM lesions, and the evaluation of BMs without a known history of primary cancer.

Although most diagnostic situations are based on advanced neuroimaging, a definitive diagnosis can be made only by histopathologic analysis, which may not be a feasible option in all cases. Consequently, a high clinical suspicion for BMs, in the context of an active or recently diagnosed primary cancer, can justify management without conditional histopathologic confirmation.

### Imaging

A brain magnetic resonance imaging (MRI) study with contrast is the method of choice for the diagnosis and primary assessment of BMs [41]. The typical radiographic appearance of BM lesions, with contrast application, is circumscribed, enhancing masses with cystic or mixed (cystic and solid) features, surrounded by disproportional vasogenic edema, and lesions are often located at the junction of gray and white matter [4]. However, these features are not specific to BMs and can also be separately found in other primary brain tumors, space-occupying infectious processes, or even vascular insults. 

MRI is preferred over contrast-enhanced computed tomography for its sensitivity in detecting lesions, especially in challenging areas (e.g., the posterior fossa), as well as for evaluating meningeal malignancy and differentiating BMs from other CNS lesions [4,42]. The superior diagnostic performance of MRI over computed tomography is also evident in determining the number and extent of BM lesions, which is crucial for formulating the treatment approach. This is important in cancers with a known propensity for multifocal BMs, such as melanoma and lung cancer, compared with other cancers with more defined and unifocal BMs, such as kidney, breast, and colon cancers [43].

Although widely used, especially in the ED, the diagnostic yield of a brain CT scan is much less informative than that of a brain MRI. MRIs surpass CT scans in detecting smaller lesions, offering better soft tissue contrast (especially for leptomeningeal lesions), and avoiding bone artifacts [42]. Therefore, despite the frequent ordering of brain CT scans for emergent neurological symptoms in the ED, brain MRIs remain the best subsequent diagnostic tool when BM is suspected, particularly in determining the next therapeutic approach, whether it involves surgery or radiation [4]. Moreover, brain CT scans can be valuable alternatives to brain MRIs for patients with contraindications for MRI, such as those with implanted medical devices or allergies to gadolinium-based contrast agents.

Other sophisticated imaging techniques are either focused on the tumoral content (diffusion-weighted MRI) or the peritumoral environment (perfusion and metabolism-weighted imaging) [44]. Comparative characteristics of BMs and other brain tumors are summarized in Table 2. 

## 5. Management

### 5.1. Biopsy

A histopathologic diagnosis should be highly considered in two main contexts: an unknown primary tumor, and atypical cerebral lesions on imaging with a history of an active primary or long-term controlled primary tumor. 

Biopsies can change the suspected diagnosis of BM in patients with single lesions: a prospective study identified a shift of at least 10% of suspected BM diagnoses to infectious, inflammatory, and other primary neoplastic conditions [48]. Also, few tertiary centers with abundant resources will irradiate a “presumed” BM lesion without getting a stereotactic biopsy or minimally invasive surgery. Another rationale for obtaining surgical tissue is the possibility of molecular analysis, thanks to the growing understanding of molecular distinctions between BMs and extracranial tumors. Currently, routine histologic workup with immunohistochemistry helps differentiate BMs from primary gliomas and other entities [4]. The applications of genetic sequencing and the identification of drug resistance mutations in cerebrospinal fluid liquid biopsies is another promising direction of research [49].

The benefit of a biopsy can be seen among patients with unknown primary tumors. This scenario is frequently encountered in SCLC and lung adenocarcinomas, with BM cases occurring, respectively, in 15.8% and 14.4% of patients at the original diagnosis in large population-based analyses [12]. Histopathologic examination of the brain biopsy can be helpful to identify the primary tumor, which is often lung cancer (56–80% of BM cases without a known primary), followed by melanoma or colon cancer [50,51,52]. These challenging presentations often necessitate a thorough clinical investigation with chest, abdominal, and pelvic computed tomography scans, or even positron emission tomography scans, to identify the primary cancer and assess disease dissemination [50].

Patients with breast cancer with a single dural-based lesion also face a special diagnostic challenge due to the higher incidence of meningiomas in this population [53]. Imaging often proves inconclusive; however, the presence of peritumoral brain edema and irregular lesion margins can suggest a dural metastasis rather than a meningioma. In such cases, a biopsy or surgical resection of the lesion can be helpful to differentiate between these diagnoses.

When the brain tumor is accessible, a biopsy can be performed either through a minimally invasive procedure with a needle or during surgical intervention. In addition to its diagnostic role, biopsy can also help guide treatment. Even if the primary tumor has been identified, BM tissue is still important to provide clinical guidance due to molecular differences between primary and metastatic lesions [4]. Once the clinical workup is completed, treatment options will include surgical intervention, RT, and/or systemic therapies [54]. The ultimate goal of the treatment of BMs is to maximize overall survival and optimize QoL by choosing the least toxic approach among available therapies.

### 5.2. Surgery

Craniotomy stands as the primary surgical intervention in patients with an accessible and limited number of BMs, good performance status, and a controlled primary disease [55]. Surgery is also beneficial for patients with multifocal BM lesions, mainly when the lesions are symptomatic and causing mass effects, provided that the lesions can be accessed without collateral neurological deficits [56]. 

However, potential complications such as seizures, surgical wound infections, and the onset or worsening of neurological deficits must be carefully considered [57]. Moreover, surgery alone, with control rates of 50%, is not sufficient for controlling local expansion of the BM lesion; therefore, additional modalities such as postoperative RT might be necessary [58]. In fact, the combination of surgery with RT for the treatment of a single large BM lesion has shown a 1-year local control rate ranging from 69% to 100% [59]. 

### 5.3. Radiation Therapy

The standard of care for patients with limited BMs is currently surgery with postoperative RT [55]. Standalone RT is also considered for patients with an asymptomatic BM lesion or multiple BM lesions and/or those not eligible for surgery due to comorbidities or problematic locations. However, some studies have shown no significant outcome difference between standalone RT and postoperative RT regarding local disease control and overall survival [60,61,62].

Historically, whole-brain RT (WBRT) used to be the primary approach [8] because it could be initiated promptly to target visible and occult lesions [63]. The role of WBRT is valuable in cases of multiple BM lesions to improve local and distant CNS tumor control. However, the well-recognized toxicity profile of WBRT includes cognitive adverse events such as somnolence and memory impairments, as well as physical function deterioration, appetite loss, and fatigue [64]. Radiation necrosis can also occur when a healthy brain parenchyma is irradiated [65]. For these reasons, RT has recently shifted towards less toxic techniques, such as stereotactic radiosurgery (SRS) and multi-fraction stereotactic RT (MFSRT), which have shown improved cognitive preservation [66]. 

SRS delivers a targeted dose in a single session, making it the preferred approach for a limited number of BM lesions, particularly in postoperative cavity RT [64]. This RT technique is being used to target BMs near the optic nerve, brainstem, or any other sensitive brain tissue, and it has shown a better toxicity profile than that of other RT techniques. However, the risk of radiation necrosis is still present with SRS, and the risk increases with the volume of the cavity irradiated [67]. Radiation necrosis can manifest 1 to 2 years after treatment, either through radiographic changes or symptomatic presentations such as headaches, drowsiness, seizures, and potentially death [68]. 

MFSRT is a potential alternative to treat large postoperative cavities following the removal of a BM lesion [69]. This technique fractionates radiation regimens to maximize the biologically effective dose delivered to the lesion and reduce the risk of radiation necrosis in healthy brain tissue [69]. Three to five fractionated SRS sessions have also been reported in patients with multiple BM lesions [70]. MFSRT has also been commonly employed for treating lesions close to critical structures such as the brainstem [69]. Determining the exact doses of radiation delivered with MFSRT is still challenging due to the lack of prospective trials. However, in studies comparing RT techniques, significantly lower rates of radiation necrosis have been reported with MFRST (0–8%) than with SRS (13–30%) [71,72,73]. Studies have also shown that 1-year local control after MFSRT alone ranged from 65% to 96% [74,75]. Overall, MFSRT can be effective with minimal toxicity when administered postoperatively and may also be used as neoadjuvant therapy prior to surgery.

Historically, regarding overall survival, patients with uncontrolled extracranial disease did not benefit from WBRT alone [76]. Moreover, in some studies, patients with multiple accessible lesions who had surgery followed by postoperative SRS seemed to benefit more than those who received WBRT, in terms of local control of the disease [77]. This conclusion is contradicted by recent studies showing 20% less local control at the resection site following SRS compared with WBRT. This could be explained by inconsistencies in identifying the target area after a surgical resection [78]. Interestingly, in patients with controlled disease who have up to three BM lesions, the combination of SRS and WBRT has shown improved survival rates [79]. Therefore, more research is still needed to identify the most appropriate approach for each patient.

One of the several approaches proposed to reduce RT toxicity is intensity-modulated RT, which contours radiation precisely to a designated region via linear accelerators. This technique aims to spare critical structures such as the hippocampus with minimal radiation doses to healthy tissue [80]. Memantine (N-methyl-D-aspartate glutamate receptor blocker) is used to help treat vascular dementia. It has shown promising results in delaying cognitive decline and preventing glutamate-induced excitotoxicity from radiation exposure [81]. The combination of hippocampal avoidance and memantine is beneficial in patients without hippocampal metastases.

### 5.4. Systemic Therapy

#### 5.4.1. Chemotherapy and Targeted Therapy

Systemic chemotherapy and molecular targeting treatments can be used with the treatment options mentioned above, to decelerate tumor growth and postpone tumor-related symptoms. Several chemotherapies and molecular therapies have been tested for the predominant cancers associated with BMs, including NSCLC, breast cancer, and melanoma. More specifically, molecular targeted therapy has emerged as a promising adjuvant treatment to surgery or RT in the treatment of BMs.

For BMs of NSCLC, some tyrosine kinase inhibitors, such as osimertinib, which targets EGFR-mutant NSCLC [82], and alectinib or brigatinib, which target ALK-positive NSCLC [83,84], have shown better CNS penetrance and disease control than traditional systemic chemotherapy [85]. For BMs of breast cancer, combinations of the tyrosine kinase inhibitor neratinib with capecitabine chemotherapy have also shown promising results in controlling disease in HER2-positive BMs [86]. For BMs of BRAF-mutant melanoma, BRAF inhibitors such as vemurafenib and dabrafenib are active in treating BMs [87]. 

#### 5.4.2. Immunotherapy

Another treatment option for more durable management of BMs is immunotherapy. Research in this area has been promising, and the use of immune checkpoint inhibitors has been evaluated in trials, particularly in patients with primary lung cancer or melanoma [88]. Currently available immune checkpoint inhibitors predominantly consist of two types: cytotoxic T-cell lymphocyte-4 (CTLA-4) inhibitors and programmed cell death 1/programmed cell death ligand-1 (PD-1/PDL-1) inhibitors [89]. The combination of RT with immune checkpoint inhibitors seems to exhibit a synergistic effect in sustained treatment of BMs [90,91]. Further evaluation is needed to understand the mechanisms of immune checkpoint inhibitors in this specific scenario and potentially include them in the standard of care for BMs.

In a recent phase II study, Nadal et al. evaluated the combination of atezolizumab (an anti-PDL-1 immune checkpoint inhibitor) with carboplatin and pemetrexed in patients with NSCLC with a stable, untreated BM lesion. This combination showed efficacy in controlling intracranial disease, and these results support the use of upfront chemo-immunotherapy, with RT reserved as a salvage option. By deferring local therapy, patients with a clinically stable BM lesion could maintain their QoL by preventing or delaying toxicities associated with RT [92]. 

## 6. Surveillance and Screening

Imaging surveillance is critical for all patients treated for BMs. The European Association of Neuro-oncology and European Society for Medical Oncology recommend neurological examination with brain MRI every 2–3 months in patients with known BMs, or when neurological progression is suspected. Also based on these guidelines, subgroups of cancer patients with an increased risk of BMs may benefit from neuroimaging screening via MRI at the time of primary cancer diagnosis. These subgroups include patients with stage IV melanoma, stage IV breast cancer (either triple-negative or HER2-positive), or lung cancer (with the possible exception of stage I NSCLC). These screenings can help in the early detection of BMs, leading to potentially better disease control [30,93].

## 7. Follow-Up

Following an ED visit, patients with cancer exhibit a higher rate of inpatient admissions compared with the general population [94]. However, not all of these patients need extended hospital stays exceeding two midnights. When home discharge with close outpatient monitoring is unavailable, observation unit visits may be useful. In fact, observation visits are increasingly supplanting brief hospital stays among Medicare patients [95].

Patients with cancer typically report better QoL when symptoms are managed at home and seek to reduce hospitalization to ensure their comfort and well-being. The risk of nosocomial infections is also reduced with a shorter hospital stay, which is particularly desirable for this category of patients [96].

However, the presence of BMs often signals advanced systemic disease, and these cancer patients usually present to the ED with poorly controlled symptoms, justifying escalated care and hospitalization [97,98]. Half of the patients with cancer who present to the ED report pain, fever, dehydration, nausea, and emesis, among other symptoms. These symptoms are identified by the quality metric of Medicare and Medicaid Services as potentially manageable in the outpatient setting, ultimately reducing ED use and hospital admissions. Therefore, an improved understanding of the reasons for potentially avoidable hospitalizations is needed [5].

Another possible reason for hospitalization is the inconsistency of care providers. Cancer patients with BMs typically have complex medical histories, which are managed by specialized oncologic teams. Yet, these patients often present at local EDs with no clear prior records, which may prompt emergency physicians to conduct further investigations and potentially admit the patients [99,100].

Over the last two decades, BM management has evolved and continues to transform. Patients diagnosed with BMs frequently face diminished QoL and complex decision-making processes due to numerous treatment options and limited prognostic information. Planning the future with patients is crucial to ensure that care aligns with their goals. This includes discussions about hospitalization, ICU stays, and reviewing end-of-life preferences and advanced care plans upon diagnosis of BMs [101]. These decisions demand a delicate balance between assessing intervention and symptom management options [102]. Reducing ED use will require more aggressive and preventive management of expected complications among cancer patients. Providing acute access to medical support for cancer patients who may not require ED care or hospital admission will be a major challenge for healthcare providers [5].

Studies describing the use of ED and hospital admission among cancer patients with BMs are lacking, and the reason for the escalation to inpatient hospital-level care remains unclear. Leading such studies could empower emergency physicians to identify cancer patients with BMs who can be safely discharged and benefit from outpatient follow-up, including those who require an initial hospitalization in an observation unit or need inpatient admission [103,104].

One remaining challenge in modern oncology is the process of shared decision-making with patients. Supporting patients in their cancer journey requires clinicians to allow them to express their emotions and understanding. The ask/tell/ask model is effective: ask what patients know, what they want to know, and if they are ready to continue the conversation before delivering additional information. This method enables clinicians to communicate information clearly and empathetically and strengthens the clinician–patient relationship, ensuring optimal care regardless of the outcome [101].

## 8. Conclusions

Despite the poor prognosis associated with BMs, successful palliative care remains an important target, with more patients achieving longer periods of controlled disease [2]. Effective management of BMs requires achieving local control while safeguarding healthy brain parenchyma and reducing therapy-related side effects. Neuroimaging findings, histologic characteristics of the brain lesion (if available), the patient’s general condition, and prior treatment regimens should guide the management of BMs. Surgical resection followed by cavity RT or definitive SRS/MFSRT is the standard approach, depending on the primary disease stage and patient comorbidities. Emerging targeted therapies hold promise when combined with current options, but the use of targeted therapies is limited to specific patient populations. Hence, the ultimate goal for cancer patients is to achieve a better QoL, ideally with a minimal hospital stay. However, managing BMs remains a challenge, particularly in the ED setting. There are no clear guidelines regarding the post-ED course of cancer patients with BMs and, specifically, whether they should be admitted, observed, or can be safely discharged.

Further investigations are needed to develop new recommendations that could improve the QoL of cancer patients with BMs by adequately controlling disease progression.

## Figures and Tables

**Table 1:** Management of common symptoms in patients with brain metastases in the emergency department.

Symptom	Management
**Peritumoral edema**	
**Mild symptoms** (e.g., headache)	Dexamethasone 2–4 mg/day orally, 1 to 2 divided doses
**Moderate to severe symptoms ** (e.g., severe headache, vomiting, seizures, focal deficits)	Dexamethasone Loading dose 10 mg IV Maintenance 8–16 mg/day orally/IV, 1 to 4 divided doses
**Increased intracranial pressure** (e.g., confusion, drowsiness)	Dexamethasone Loading dose 10 mg IV Maintenance 16–40 mg/day orally/IV, 2 to 4 divided doses Hypertonic saline Mannitol
**Epileptic seizure**	Levetiracetam 2–250 mg/day (first week), then 2–500 mg/day Therapeutic plasma range: 5–25 mg/L (and/or) Valproic acid 20–25 mg/kg per day Therapeutic range: 50–100 mg/L

Abbreviations: IV, intravenous.

**Table 2:** Imaging characteristics of brain metastases (BMs) compared with other brain lesions for different imaging techniques.

Imaging Technique	Value in Diagnosis
CT scan of the brain	Less sensitive than MRI for BM detection (less soft tissue contrast and more bone artifacts), generally reserved for patients with contraindications for MRI [4,42]
Diffusion-weighted MRI	Less restricted diffusion in BMs compared with pyogenic abscesses [44]
Perfusion-weighted MRI	Lower cerebral blood volumes in the peritumoral area of BMs compared with that of glioblastomas; higher cerebral blood volumes in enhancing rims of BMs compared with those of pyogenic abscesses [45] Generated time–signal curves can differentiate BMs from primary lymphomas and gliomas [46]
T2-weighted and FLAIR MRI sequences	Better visualization of disproportional vasogenic edema in BMs (more signal intensity), despite differences in all BM edemas [4]
Magnetic resonance spectroscopy	Lower choline/creatinine ratios in BMs compared with high-grade gliomas [47]
FDG-PET	Difficult to differentiate BMs from high-grade gliomas (due to high glucose uptake in the brain overall), more useful in extracranial non-parenchymal metastasis [45]

Abbreviations: CT, computed tomography; MRI, magnetic resonance imaging; PET, positron emission tomography.
